# Mapping the association between tau-PET and Aβ-amyloid-PET using deep learning

**DOI:** 10.1038/s41598-022-18963-6

**Published:** 2022-08-30

**Authors:** Gihan P. Ruwanpathirana, Robert C. Williams, Colin L. Masters, Christopher C. Rowe, Leigh A. Johnston, Catherine E. Davey

**Affiliations:** 1 Department of Biomedical Engineering, The University of Melbourne, Melbourne, VIC Australia; 2 Melbourne Brain Centre Imaging Unit, The University of Melbourne, Melbourne, VIC Australia; 3 Department of Molecular Imaging and Therapy, Austin Health, Melbourne, VIC Australia; 4 Florey Institute of Neuroscience and Mental Health, Melbourne, VIC Australia; 5 Florey Department of Neuroscience and Mental Health, The University of Melbourne, Melbourne, VIC Australia

**Keywords:** Alzheimer's disease, Prognostic markers

## Abstract

In Alzheimer’s disease, the molecular pathogenesis of the extracellular Aβ-amyloid (Aβ) instigation of intracellular tau accumulation is poorly understood. We employed a high-resolution PET scanner, with low detection thresholds, to examine the Aβ-tau association using a convolutional neural network (CNN), and compared results to a standard voxel-wise linear analysis. The full range of Aβ Centiloid values was highly predicted by the tau topography using the CNN (training *R*^2^ = 0.86, validation *R*^2^ = 0.75, testing *R*^2^ = 0.72). Linear models based on tau-SUVR identified widespread positive correlations between tau accumulation and Aβ burden throughout the brain. In contrast, CNN analysis identified focal clusters in the bilateral medial temporal lobes, frontal lobes, precuneus, postcentral gyrus and middle cingulate. At low Aβ levels, information from the middle cingulate, frontal lobe and precuneus regions was more predictive of Aβ burden, while at high Aβ levels, the medial temporal regions were more predictive of Aβ burden. The data-driven CNN approach revealed new associations between tau topography and Aβ burden.

## Introduction

Alzheimer’s disease (AD) is characterized by the accumulation of two proteins in the brain that pre-date the clinical onset of symptoms by several decades^1–5^: extracellular Aβ-amyloid (Aβ) plaques that initiate in the neocortex and gradually spread through the brain, and intracellular tau neurofibrillary tangles, which are most evident in the entorhinal cortex and the limbic system^6–8^.

Positron emission tomography (PET) tracers developed for imaging of aggregated Aβ have more recently been complimented by the development of tau radiotracers, enabling comprehensive studies that investigate topographical changes of both tau and Aβ across different stages of the natural progression of AD^3,5^. The pathogenic mechanisms by which Aβ and tau accumulate remain largely unknown^1^, and the development of Aβ-PET and tau-PET tracers have the potential to provide critical insights into their interaction over time. Earlier studies concluded that typically Aβ is deposited in cortical regions, followed by significant tau accumulation^9–12^, initially in the medial temporal lobe and latterly spreading throughout the neocortex.

Aβ-PET images are typically mapped to a scalar Centiloid (CL) value to quantify neocortical Aβ burden, which may be indicative of downstream pathological changes^13–15^. However, a comparable transformation for tau-PET images to a scalar value is not yet available.

Convolutional neural networks (CNNs) are deep learning networks designed for the analysis of image data, inspired by the concept of receptive fields in the visual cortex. The high accuracy of CNNs in mapping input images to a specified output, in conjunction with an abundance of medical imaging data, have promoted their broad application from diagnostics to image reconstruction^16–18^ and, more recently, to AD^19–23^ research. However, the complex structure of CNNs, with many layers of thousands of learned parameters, render the interpretation of this mapping difficult^24^. Recent research has employed *attribution* techniques to aid interpretation, which identify regions of the input image that are most responsible for estimating the output^25–28^. Saliency maps are an attribution technique, employing a gradient-based method of generating weights for each input voxel that denote its contribution to a specific CNN-generated output. Unlike other interpretation techniques, saliency maps are not dominated by input values, but rather depend primarily on the learned network parameters^29^.

Previous cross-sectional studies examining the interaction between Aβ and tau were principally based on linear, voxel-wise or region-of-interest (ROI), techniques. Such models did not seek to capture spatial dependencies between voxels or brain regions. Notable exceptions are studies that employ independent component analysis to examine how spatial patterns of tau change with Aβ burden^11,30^. Given that tau accumulation has strong spatial dependencies^30–33^, it is important to employ models that can accommodate these relationships. Analysis methods that can probe multivariate, nonlinear relationships between Aβ and tau across spatially remote brain regions may provide new insights into the interactions between these two molecular species. CNNs have this capability due to their multi-layered structure; a CNN passes information from input images to predicted output via a series of layers of artificial neurons. In each layer, a receptive field defines the image region over which to relate input information to output values. The size of the receptive field widens with depth in the network, thus integrating spatial dependence between input image regions in the output prediction. Moreover, the neuron activation functions permit nonlinear mappings from input to output, enabling the capture of a much broader range of relationships between Aβ and tau than voxel-wise or ROI-based linear models.

**Figure 1:**
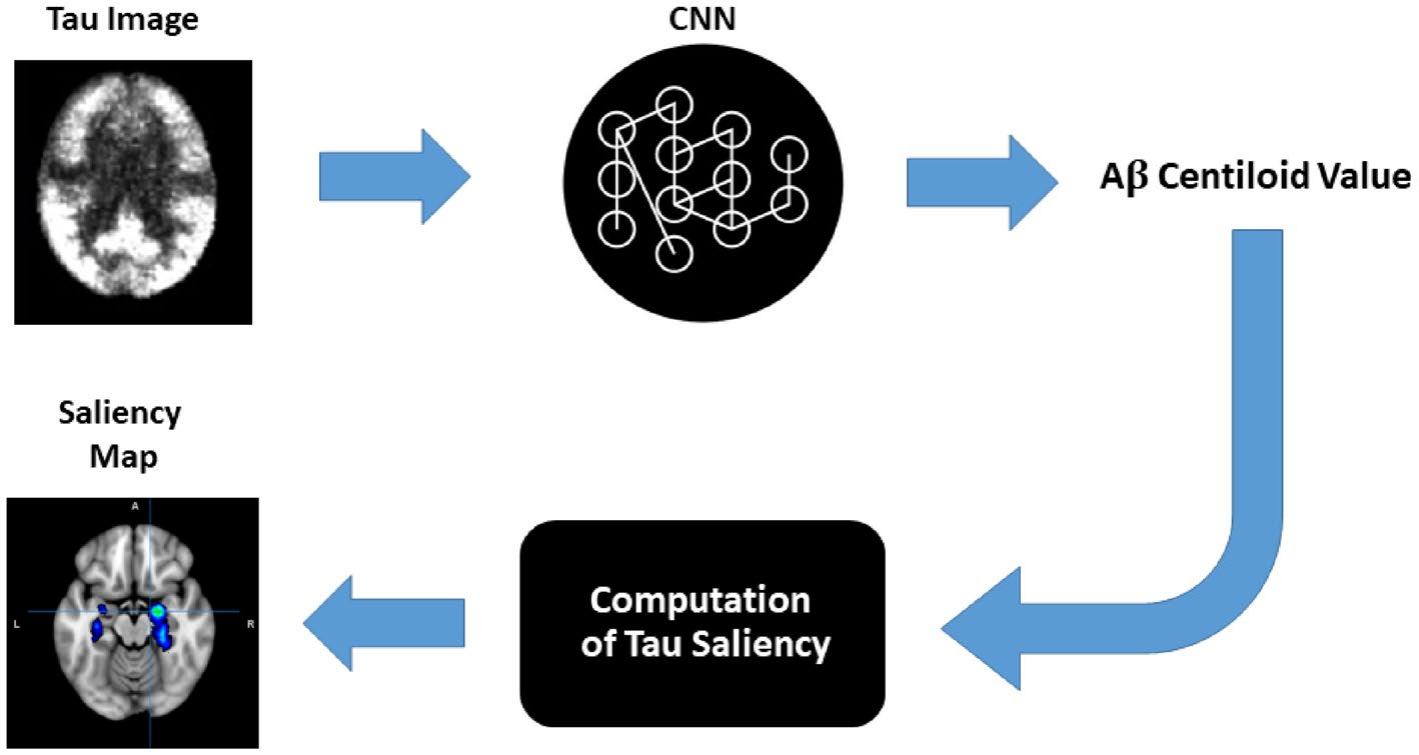
Processing pipeline for a convolutional neural network (CNN)—a deep learning technique developed to process images—to determine the association between tau-PET topography and Aβ CL. Tau images and Aβ CL values form the input and output of the CNN, respectively. After training the CNN, a saliency map is generated for every subject and associated tau-Aβ CL mapping, in which every voxel is allocated a weight to indicate the importance of its tau uptake value in the estimation of the CNN estimated Aβ CL.

In this paper, a CNN is used to examine the relationship between tau-PET images and Aβ-PET quantification across the AD continuum. [^18^F]MK6240 scans were used as input to the CNN, while Aβ CL was used as the scalar output, such that the CNN was trained to map tau images to Aβ CL. We interpreted the importance of each tau voxel in this mapping using saliency (Fig. 1). Crucially, the end-point of this study was not solely the prediction of Aβ CL from a tau-PET image, but rather to provide insight into the mapping between tau topography and Aβ CL.

## Results

In this study, we examined the relationship between tau-PET images and Aβ CL using a CNN. Demographic data for the 134 subjects used in this analysis are shown in Table 1. Missing demographic data is denoted by a ^*^. Since the data were divided into training, validation and testing datasets during the CNN implementation, demographics were calculated for each separately. Although percentages of females to males were similar in the training and testing datasets, the validation dataset had a smaller proportion of females. Subjects in the test dataset were, on average, older than those in both the training and validation datasets. Each dataset had a similar percentage of Aβ+ subjects, whilst the validation dataset had the highest percentage of Apolipoprotein E4 (APOE4) carriers.

**Table 1:** Dataset demographics with format ‘percentage (number of samples)’.

	All data	Training data	Validation data	Testing data
Sample size	134	109	12	13
Sex. F (%)	67 (50)	57 (52)	4 (33)	6 (46)
Age ± SD	70.55 ± 0.5	70.12 ± 0.5	68.83 ± 0.49	75.77 ± 0.52
% Right hand (samples)	88 (107)*	91 (85)*	67 (9)*	85 (13)*
% APOE4 (samples)	50 (98)*	48 (79)*	67 (9)*	50 (10)*
% Aβ+	49	49	50	54
CU, MCI, AD % Aβ+	40, 40, 39* (31, 58, 79)	27, 35, 33* (31, 58, 79)	5, 4, 3 (20, 57, 75)	9, 1, 3 (44, 0, 100)
MMSE ± SD (samples)	25.51 ± 2.85 (109)*	25 ± 2.72 (87)*	24.67 ± 3.08 (9)*	28.23 ± 2.09 (13)
CDR ± SD (samples)	0.5 ± 0.2 (109)*	0.55 ± 0.15 (87)*	0.56 ± 0.17 (9)*	0.19 ± 0.25 (13)
CDR SoB ± SD (samples)	2.14 ± 1.68 (109)*	2.3 ± 1.61 (87)*	2.5 ± 1.94 (9)*	0.81 ± 1.48 (13)

Missing subject data is denoted by a *. An Aβ CL threshold of 25 was used to assign subjects to Aβ− and Aβ+ groups.

*F* female, *SD* standard deviation, *APOE4* apolipoprotein E4, *CU* cognitively unimpaired, *MCI* mild cognitive impairment, *AD* Alzheimer’s disease, *MMSE* mini-mental state examination, *CDR* clinical dementia rating, *CDR SoB* clinical dementia rating scale sum of boxes.

### CNN training

As CNN performance is sensitive to network structure and parameter configuration, several different CNN models were compared. Ten-fold cross-validation was employed, so that ten instances of each model were learned. The best-performing model was identified by calculating the average performance across the ten folds, where performance was evaluated using the root mean squared error (RMSE) between measured Aβ-PET CL, and CNN estimated CL. The best-performing CNN model successfully learned to associate spatial features in tau images with Aβ CL (Supplementary Table S1), though with varied performance across the ten folds (Supplementary Table S2), reflected by the high standard deviation in Supplementary Table S1.

**Figure 2:**
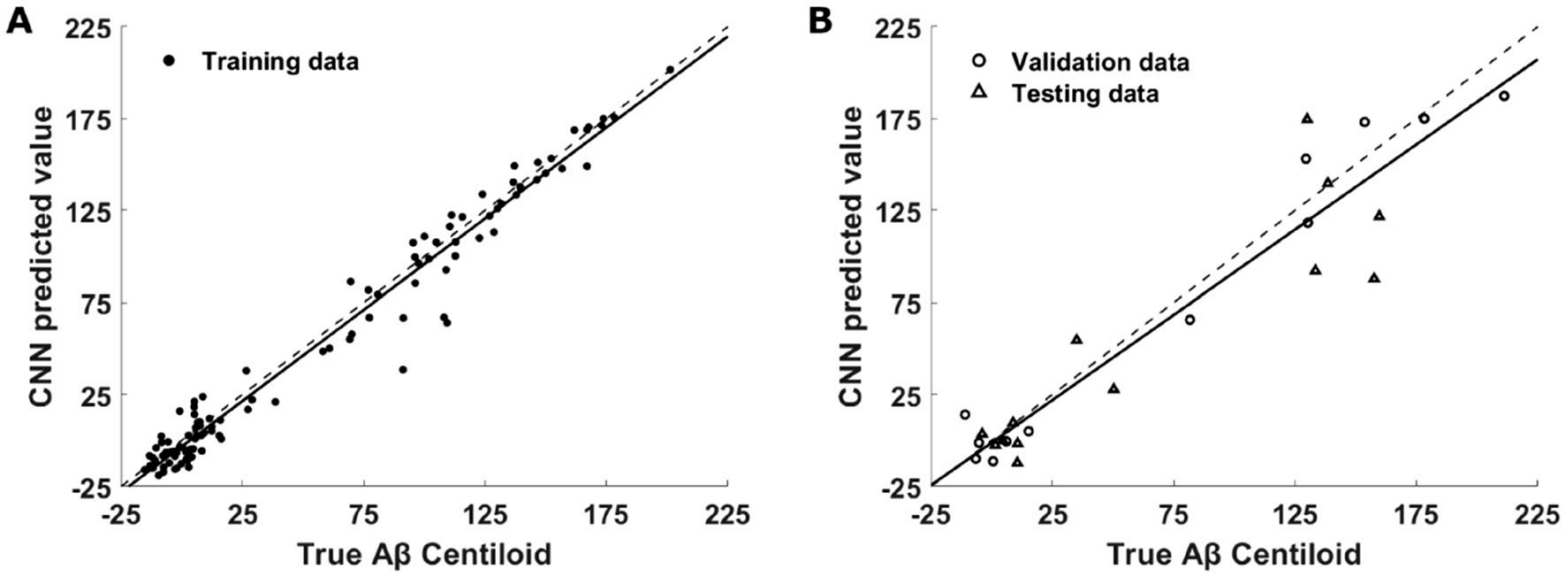
Scatter plot of measured Aβ CL values vs CNN predicted values on (**A**) training dataset (*R*^2^ = 0.97, filled dots) and (**B**) validation dataset (*R*^2^ = 0.96, open circles) and testing dataset (*R*^2^ = 0.79, triangles) for the best cross-validation instance. The solid black line is the line of best fit. The dashed black line is the ideal expected fit.

The CNN model learned in the sixth during the cross-validation fold demonstrated the best RMSE between measured and estimated Aβ CL, across both validation and test datasets, and showed consistency in performance across training (RMSE = 11.72, *R*^2^ = 0.97) and validation (RMSE = 15.08, *R*^2^ = 0.96) datasets. Furthermore, the model produced an accurate mapping for training and validation subjects across the full range of Aβ CL values (Fig. 2A,B open circles). The optimal CNN model instance produced an RMSE of 29.93 and *R*^2^ of 0.79 for the hold-out test data. While lower than the validation and training *R*^2^ values, a strong relationship between actual and estimated Aβ CL was maintained. Importantly, in the testing phase, the model continued to successfully discriminate the full range of Aβ CL values (Fig. 2B triangles). Therefore, this instance of the optimal CNN model was used to analyse tau spatial features associated with changes in Aβ burden and was compared with the standard linear, voxel-wise analysis.

A saliency map is an attribution image in which a voxel’s value indicates the importance of the voxel’s input tau value in the CNN mapping to CL output. Saliency maps were generated for each subject, and a general linear model (GLM) was used to identify voxels with a significant linear association between saliency and CL. Salient clusters were distinguished by requiring significant voxels to be part of a cluster of at least 200 voxels. This analysis was performed on all cross-validation CNN instances of the optimal CNN model, to examine consistency in the learned CNN mappings from tau to CL, to rule out overfitting of the CNN to input noise. The results show markedly consistent and overlapping regions across the ten learned CNN instances, including the bilateral medial temporal lobes, precuneus, middle cingulate, frontal lobes and paracentral lobules (Supplementary Fig. S2). Importantly, although cross-validation instances produced varied performance across the test, validation and training datasets, all CNN instances consistently identified similar salient regions in the mapping from tau to Aβ CL, irrespective of the partitioning of the data. This suggests that the learned CNNs have not overfitted to noise.

### Comparison between tau-SUVR and saliency analyses

**Figure 3:**
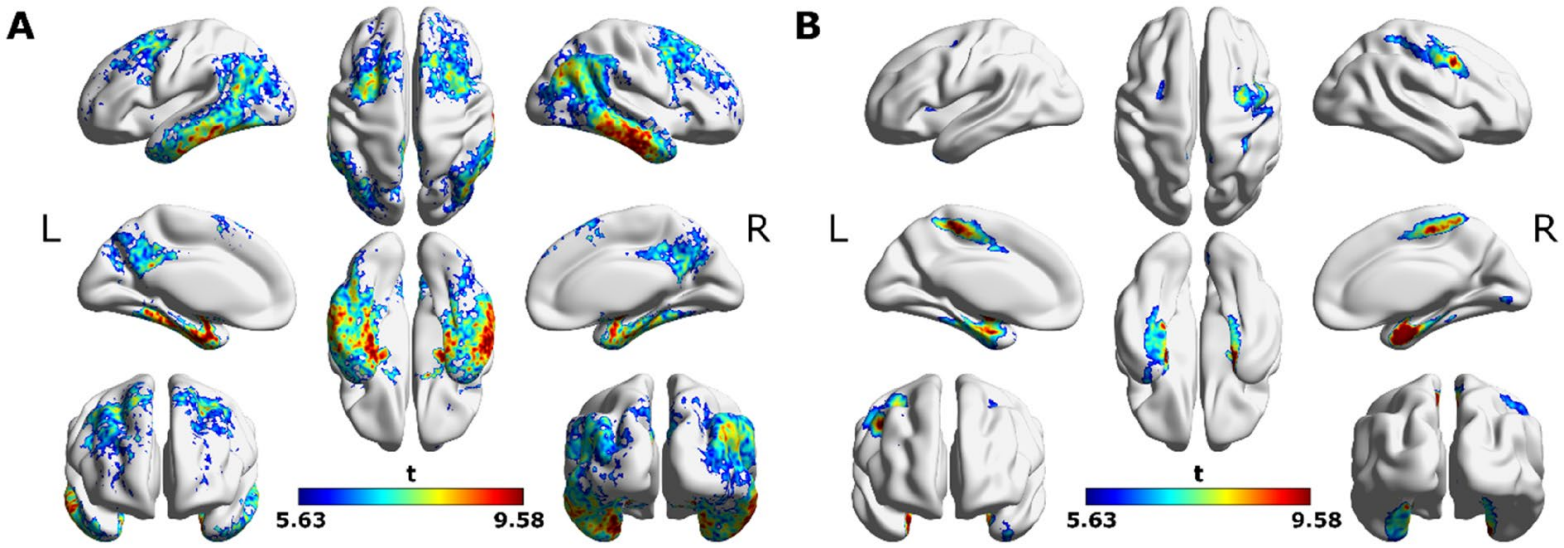
Comparison of SUVR-based and CNN-based GLM analyses on semi-inflated brain surfaces. (**A**) Significant clusters identified from voxel-wise GLMs to estimate tau-PET SUVR values from Aβ CL values (FWE *p* < 0.05; cluster extent of 200; covaried for age and sex) and (**B**) Significant clusters identified from voxel-wise GLMs to estimate CNN-based saliency values from Aβ CL values (FWE *p* < 0.05; cluster extent of 200; covaried for age and sex). L and R denote the left and right sides of the brain.

**Figure 4:**
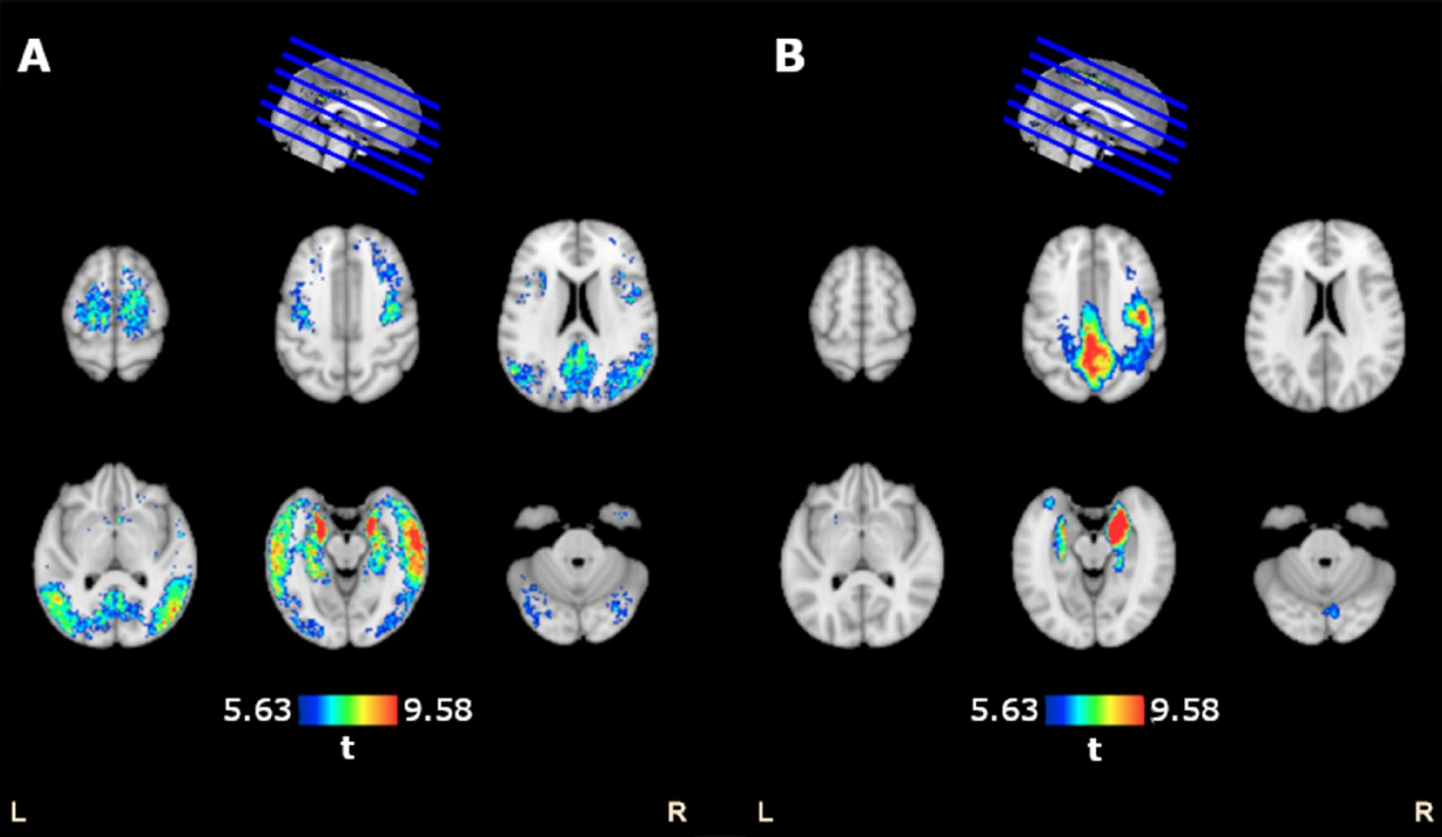
Comparison of SUVR-based and CNN-based GLM analyses. (**A**) Tau-PET SUVR-Aβ CL GLM significant clusters (FWE *p* < 0.05; cluster extent of 200; covaried for age and sex) and (**B**) CNN-based saliency-Aβ CL significant clusters (FWE *p* < 0.05; cluster extent of 200; covaried for age and sex).

To compare our CNN approach with standard linear, voxel-wise modeling, a GLM was fitted to each voxel’s tau, converted to SUVR, and Aβ CL. It identified three significant tau clusters associated with Aβ CL (Figs. 3A, 4A), which were spread across the hippocampi, parahippocampal gyri, temporal lobes, parietal lobes, occipital lobes, frontal lobes and cingulate of both hemispheres.

A GLM was used to evaluate the voxel-wise relationship between saliency maps, generated by the best cross-validation instance, and Aβ CL. It revealed five salient clusters associated with Aβ CL (Figs. 3B, 4B): relatively large clusters in the frontal lobes, precuneus, postcentral gyri, paracentral lobules, middle cingulate, precentral gyri, supplementary motor areas (SMAs) and medial temporal structures of both hemispheres; smaller clusters in bilateral occipital lobes and insula of the left hemisphere. Detailed anatomical regions are given in Table 2.

**Table 2:** Occlusion analysis.

Cluster number	Label	Areas	Post-occlusion R^2^ (training)	Post-occlusion R^2^ (validation)	Post-occlusion R^2^ (testing)
	AC	All clusters	0.16	0.02	− 0.05
1	MTR	Right hippocampus, right parahippocampal gyrus, right amygdala, right fusiform	0.51	0.43	0.15
2	FPC	Bilateral frontal lobe, precentral gyri, supplementary motor areas, paracentral lobules, superior & inferior parietal lobules, precuneus, postcentral gyri & middle cingulate	0.84	0.84	0.64
3	MTL	Left hippocampus, left parahippocampal gyrus, left amygdala, left fusiform	0.84	0.74	0.59
4	OL	Lingual gyri, calcarine sulci	0.96	0.95	0.80
5	IL	Left Insula	0.97	0.96	0.79

Correlation between measured and CNN-estimated Aβ CL values for training (*n* = 109), validation (*n* = 12) and test (*n* = 13) datasets, shown before and after removal of salient clusters. The original *R*^2^ values before the occlusion were 0.97, 0.96 and 0.79 for training, validation and test datasets, respectively.

The tau-SUVR GLM analysis captured larger clusters spread throughout the brain (Figs. 3A, 4A), the saliency maps identified smaller, more focused, clusters (Figs. 3B, 4B). In summary, although larger tau clusters were related to Aβ CL, for the CNN, sub-regions were more informative for mapping to Aβ CL.

The GLM analysis of saliency maps highlighted regions that were not captured by the tau-SUVR GLM analysis, including regions in the middle cingulate and postcentral gyri (second slices of Fig. 4A,B). Therefore, CNN used unique regions, which were not identified by the tau-SUVR analysis, for mapping to Aβ CL.

### Changes in the association between tau and Aβ CL

Occlusion analysis was used to identify the importance of salient tau clusters in the mapping to Aβ, and how this association changes across the CL continuum. The tenet of occlusion analysis states that a cluster that is more important for the estimation of a given Aβ CL will prompt a larger reduction in estimation accuracy after it is removed from the CNN input space. This change in accuracy can be averaged across the whole Aβ CL continuum by calculating the *R*^2^ value between measured Aβ CL and CNN-estimated Aβ CL, after occlusion*,* The five salient clusters identified as changing significantly with Aβ were occluded sequentially from the optimal CNN model, and the change in ${R}^{2}$ noted.

**Figure 5:**
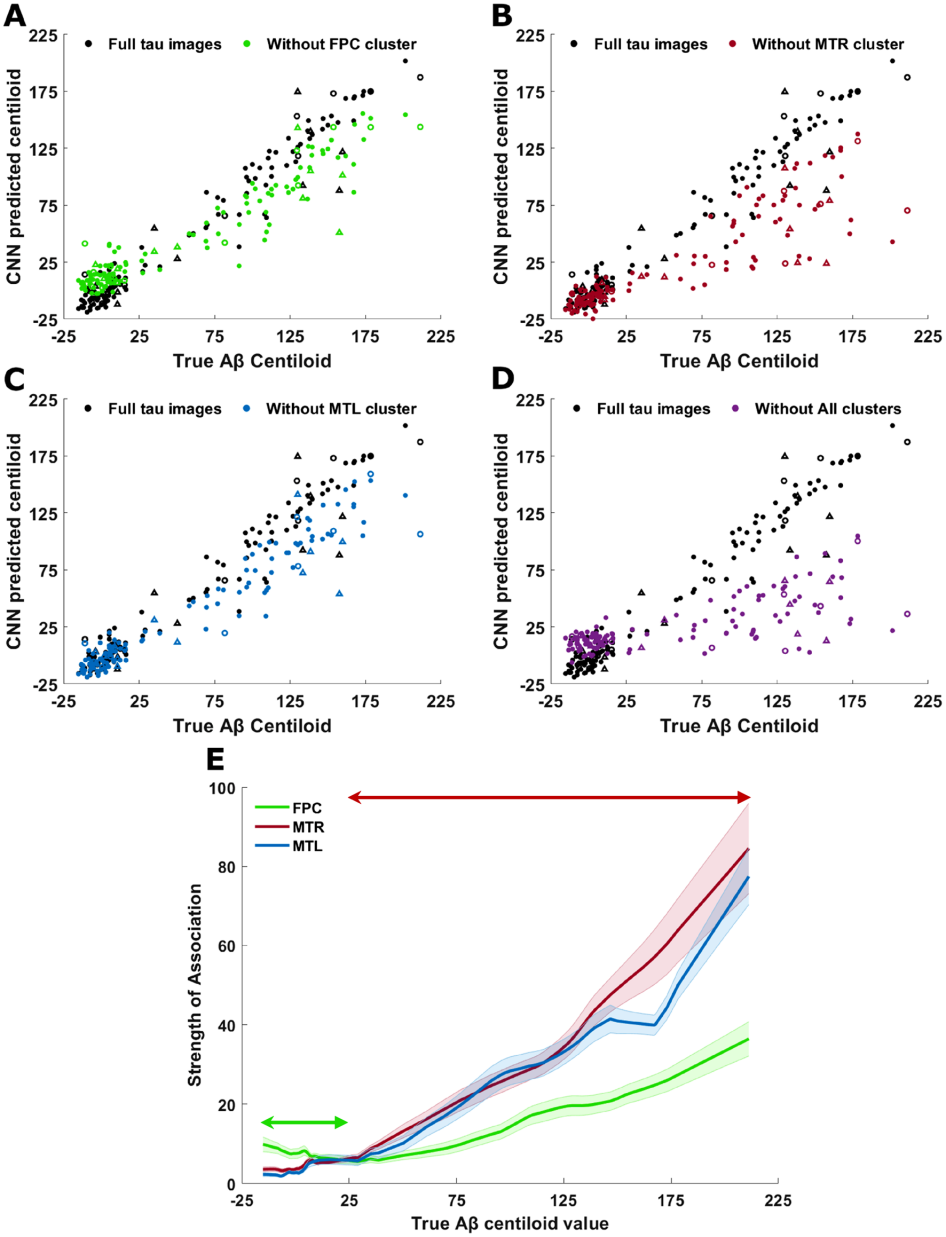
Actual versus predicted Aβ CL after occlusion of salient clusters, or clusters with saliency values that are significantly linearly associated with Aβ CL. Panels show CNN estimation of Aβ CL after occlusion of: (**A**) FPC cluster (training *R*^2^ = 0.84, validation *R*^2^ = 0.84 and testing *R*^2^ = 0.64), (**B**) MTR cluster (training *R*^2^ = 0.51, validation *R*^2^ = 0.43 and testing *R*^2^ = 0.15), (**C**) MTL cluster (training *R*^2^ = 0.84, validation *R*^2^ = 0.74 and testing *R*^2^ = 0.59), (**D**) All clusters (training *R*^2^ = 0.16, validation *R*^2^ = 0.02 and testing *R*^2^ = -0.05). (**E**) Mean strength of association of FPC, MTR and MTL tau clusters with Aβ CL, overall 10-fold cross-validation instances. Filled dots = training dataset, open circles = validation dataset, open triangles = test dataset. Shaded areas show the standard errors of the mean across the 10 folds. The green arrow denotes the Aβ CL range in which the FPC cluster is the most important, having the highest strength of association, in the CNN prediction. The red arrow denotes the dominance of MTR and MTL clusters in CNN prediction at high Aβ CL values.

Of the five occluded clusters, removal of FPC, MTR and MTL resulted in the largest reduction in *R*^2^ (Table 2). Figure 5A–C demonstrates these effects graphically, with a rotation of the scatter plots towards the x-axis after occluding the clusters from the input space, indicating reduced accuracy in the estimation of Aβ CL. Figure 5B,C establishes the importance of both left and right medial temporal structures in the CNN estimation of Aβ CL (Table 2). Occlusion of the right medial temporal lobe, denoted MTR, caused the biggest reduction in *R*^2^ (Fig. 5C).

When all clusters were occluded from the input, the predictive capacity of the CNN was reduced for the training, validation and test datasets to an *R*^2^ of 0.16, 0.02 and − 0.05, respectively (Table 2, Fig. 5D). This suggests that most of the CNNs predictive capability is captured in these five clusters.

We introduce a metric entitled ‘strength of association’ to evaluate the change in the importance of an identified cluster across the Aβ CL continuum. The strength of association is calculated by smoothing the absolute errors between CNN outputs corresponding to full tau input images and occluded input tau images. In order to reduce the bias from the best cross-validation instance, this metric was calculated for best instance clusters using all the cross-validation instances to calculate the mean strength of association for the CNN model. At low Aβ CL values, the FPC cluster had a higher strength of association than MTL and MTR clusters, that gradually decreased with increasing Aβ CL until an Aβ CL of approximately 25 (Fig. 5E, double-ended green arrow). At this point, the MTR and MTL began to dominate the mapping in all cross-validation models, becoming increasingly important in the CNN mapping of high CL values (Fig. 5E, red arrow). In summary, the CNN network used more tau information from the FPC and both MTR and MTL to predict Aβ CL in low and high Aβ subjects, respectively.

**Figure 6:**
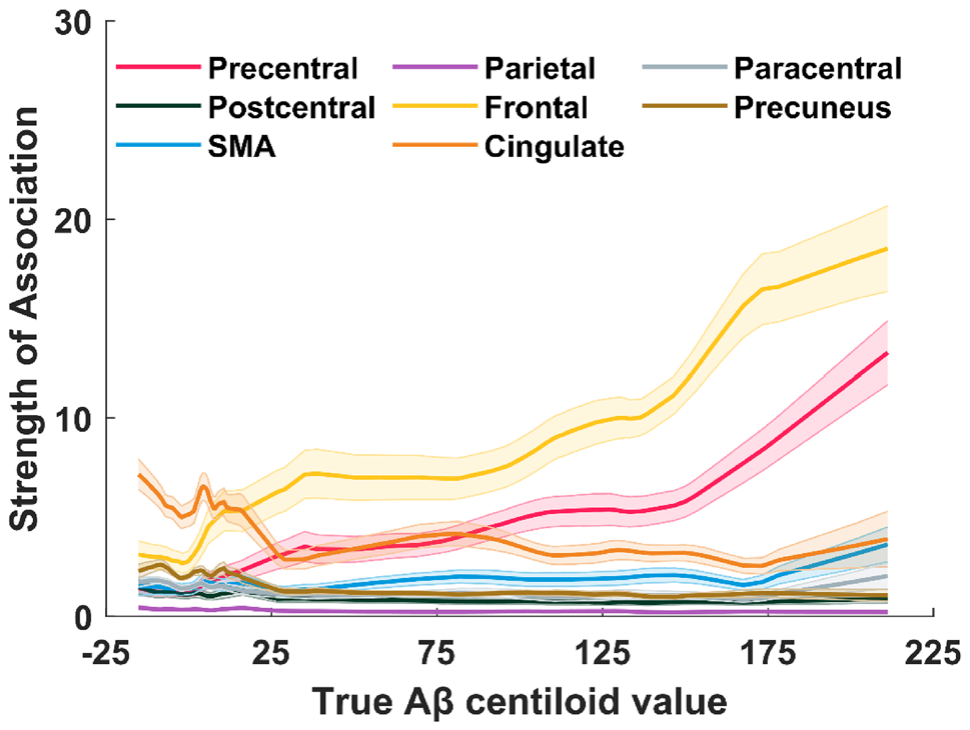
Strengths of association of sub-clusters of FPC cluster against Aβ CL value. The mean strengths of association of these sub-clusters were calculated over all cross-validation instances. Shaded areas show the standard errors of the mean lines.

Since the FPC is a large cluster, containing more voxels than any other cluster and demonstrating high strength of association across the Aβ CL continuum, sub-clusters of the FPC were sequentially occluded from the CNN input space to gain more insight into the impact of the FPC on Aβ CL estimation (Fig. 6). Sub-clusters in the cingulate, precuneus and frontal lobe had the largest strength of association at low Aβ CL, with the cingulate and precuneus gradually decreasing in strength with increasing Aβ CL. Conversely, the frontal lobe region showed an increasing strength of association with increasing Aβ CL. Other sub-clusters of the FPC, spread in the parietal lobe, paracentral lobule, postcentral gyrus and SMA, remained at low strengths across the Aβ CL continuum, except the precentral gyrus, that showed an increasing association over Aβ CL, to become one of the strongest sub-clusters at Aβ CL values of 25 and above.

## Discussion

In this study, we examined the relationship between tau-PET images and the Aβ burden using a CNN. The association between tau accumulation and Aβ burden has been investigated in several studies^9–12,32,34–37^. However, they were carried out with an assumption of spatial independence in tau accumulation across brain regions. Such an assumption is in contradiction to the growing body of research establishing the spatial dependence of tau accumulation in the natural progression of AD^30–33^. The use of a CNN, designed to extract spatial features and learn dependencies between spatially remote brain regions, enabled us to relax this assumption. Moreover, CNN analysis is a data-driven approach without the assumption of a particular spatiotemporal evolution of tau^6^; recent research has suggested the importance of the re-examination of tau topographic staging due to its heterogeneity^38^.

The relationship between Aβ and tau is often examined by separating patient data into Aβ^–^ and Aβ^+^ groups, thresholded using Aβ burden; the capacity of a CNN to identify non-linear mappings between Aβ and tau allowed us to treat Aβ quantification as a continuum, avoiding both binarizing subjects into two groups and a priori assumptions regarding the threshold. Rather, the use of a CNN enabled an exploration of associations between tau and Aβ without such preconceptions.

In our exploration of the relationship between the [^18^F]MK6240 uptake pattern and Aβ CL value, both tau-SUVR voxel-wise linear models and a CNN based nonlinear model were implemented, mapping tau-PET voxels to Aβ CL values to examine the potential of CNN models to delineate the association between these two molecular species. A voxel-wise GLM on tau-SUVR identified a positive correlation between tau accumulation and Aβ CL value throughout the brain after controlling for age and sex. In contrast, the CNN-based model identified focal clusters in the medial temporal, precentral gyri, postcentral gyri, SMAs, paracentral lobules, superior and middle frontal gyri, precuneus, superior and inferior parietal lobules, and middle cingulate, as the regions in which information from the tau scan was most salient for predicting Aβ CL value. This was elucidated by the removal of these clusters resulting in significantly reduced predictive power of Aβ CL value from tau-PET images, demonstrating that the small clusters carry more information to predict reliably Aβ burden. A possible explanation for these focal clusters is that as CNNs integrate spatial dependence between input image regions in predicting the output, they may not use duplicate information from brain regions while generating the Aβ CL.

The CNN-based nonlinear mapping between tau-PET and Aβ CL values produced markedly different topographic patterns from those emerging from the linear tau-SUVR analysis. Although the medial temporal and some frontal lobe structures were common to both saliency-based CNN analysis and tau-SUVR analysis, the CNN used information from additional brain regions, including regions in the middle cingulate and postcentral gyri, associated with the Aβ burden that was not captured by tau-SUVR analysis. Further investigation is required to determine why the CNN model identified these specific regions as important for the prediction of Aβ CL.

Our GLM analysis of tau-SUVR images showed that the level of tau accumulation in medial temporal lobes is linked with Aβ CL value, a region postulated as an initial tau accumulation site during AD evolution^6,34^. This association was not limited to the medial temporal lobes, with spread throughout the brain showing a pattern similar to Braak staging, with initiation in medial temporal structures that spreads to the neocortical regions. These results show the level of tau accumulation not only in medial temporal lobes, but also in other cortical regions, is related to Aβ burden as seen in previous studies^9–12^.

The CNN model was used to examine how changes in tau topography are associated with changing global Aβ burden. We employed occlusion analysis, in which salient clusters were sequentially occluded from the input space, and the resultant impact on the CNN estimation of Aβ CL was determined. Occlusion analysis indicates the uniqueness of information provided by the occluded region—if the CNN estimation of Aβ CL is significantly altered, it suggests that the occluded region is providing information not available from other input clusters. Additionally, the strength of association metric that was introduced to quantify changes in CNN Aβ CL estimation accuracy does not make any assumption of linearity between input tau values the Aβ CL. The strength of association measures clearly demonstrate non-linear mappings between input tau values and Aβ CL estimates, with the relative importance of clusters changing across the continuum.

In this study, we considered both Aβ and tau as continuous variables, avoiding the standard dichotomous paradigm. As per the CNN analysis, at low Aβ levels, tau clusters in the frontal lobes, parietal lobes and cingulate (FPC cluster) were more associated with Aβ burden, driven primarily by sub-clusters in the cingulate, precuneus and frontal lobe. At high Aβ levels, information from both medial temporal regions was most prominent in the prediction.

The variable importance of brain regions at different Aβ CL values provides a proxy for longitudinal studies that show temporal changes of tau topography with the Aβ burden. Aβ accumulation initiates in the precuneus, superior and inferior parietal lobules, cingulate gyrus and prefrontal cortex^6,39^, and our CNN-driven approach has captured tau sub-clusters in this area that are predictive of low Aβ burden. Further, information from medial temporal regions was the most important in predicting high Aβ levels. These pseudo-longitudinal results are consistent with the local/remote hypotheses of Aβ, in which Aβ drives local and remote effects^40^. Further, The FPC cluster overlaps with regions of the default mode network, including the superior and middle frontal gyri, precuneus and inferior parietal lobule^41^.

The FPC cluster contains regions, such as the SMAs, precentral gyri and paracentral lobules that have not been shown to exhibit an elevated tau signal. However, the strength of association for these clusters remained flat at a low value across Aβ CL values, except for the sub-cluster in the precentral gyrus; further work is required to elucidate why the CNN has identified those sub-clusters in the FPC cluster.

After the occlusion of all significant salient clusters, there remains minor residual predictive power (Fig. 5D). Saliency analysis captures predictive brain regions that are common across the cohort. However, there may be salient regions unique to an individual that leaves residual predictive power after removing common salient clusters. The limited predictive power remaining after removal of common clusters suggests that the CNN analysis has identified cohort-wide regions predictive of Aβ burden. Although it is argued that tau spreads follow a stereotypical pattern, studies have shown an inter-individual tau heterogeneity, of which the residual predictive power is suggestive^33,42,43^.

As with many other deep learning applications in biomedical imaging, data scarcity is a challenge in training the network. The training dataset performance is higher than the validation dataset, which may be thought to indicate overfitting. However, the CNN has performed well on the hold-out test dataset, successfully differentiating the full range of Aβ CL subjects. Additionally, all cross-validation instances showed overlapping spatial features in bilateral medial temporal, frontal and parietal lobes. These results suggest that our model has captured spatial features of tau images that are genuinely associated with changes in Aβ CL values, and are necessary to accurately map a tau image to CL.

The limitations of the current study include: (1) clinical diagnosis classifications were not used in this study and subjects with a range of Aβ values, both Aβ+ and Aβ−, were used; (2) all conclusions were drawn on cross-sectional analysis. Longitudinal analysis is required to investigate the insights provided by this study; (3) age and sex were used as confounding variables, however, other factors may impact tau uptake, including education level and genetics; (4) this analysis was performed on a cohort with a limited number of samples that may not cover the full spectrum of AD tau patterns; (5) while the saliency technique used to interpret the CNN results can identify regions useful for predicting of Aβ CL, it cannot be used to infer any conclusion with regard to the accumulation of tau, only that tau in these regions are used by the model to drive its prediction; (6) the findings may be dependent on the chosen PET image reconstruction characteristics. To enhance the CNN analysis, we used highly converged image reconstructions with less partial volume effect that are of higher resolution and noisier than those used for clinical viewing. This may limit the reproducibility of the study if constrained to using standard clinical PET reconstruction settings.

Irrespective of the above limitations, the study has highlighted that a deep learning approach reveals distinct information to standard voxel-wise analyses. Focused tau clusters were identified that were strongly predictive of Aβ CL values. In future studies, it will be interesting to analyse the models retrained on input tau images without clusters identified from this CNN analysis. Moreover, future studies will be targeted at more generalized methods, expanding the analysis to a range of scanners, and reversing the method to see if the topography of Aβ plays a role connecting to a variety of tau measures, including blood and CSF biomarkers as the tau markers.

## Materials and methods

### Participants

Participants were drawn from the Australian Dementia Network (ADNET) study, the Australian Imaging, biomarker and Lifestyle study (AIBL) and healthy controls from the Traumatic Brain Injury (TBI) study. A total of 134 subjects, 99 from ADNET (mean age 71.57 ± 7.69 years, 50 females), 25 from the traumatic brain injury study (mean age 63.08 ± 12.60 years, 11 females) and 10 from AIBL (mean age 79.2 ± 5.41 year, 6 females), were included (Table 1). The human scans were approved by the Austin Health Human Research Ethics Committee (HREC/18/Austin/201) and all the experiments were performed in accordance with relevant guidelines and regulations.

### Image acquisition and processing

PET scans were performed on a Siemens Biograph 128 mCT PET/CT scanner at the Melbourne Brain Centre Imaging Unit, the University of Melbourne. Subjects were scanned for Aβ and tau on two different days. A low-dose CT scan was carried out prior to each PET acquisition for attenuation correction. For Aβ scans, subjects were injected with [^18^F]NAV4694 radiotracer 50 min prior to 20 min of continuous scanning. Scanning tau with [^18^F]MK6240 radiotracer used a 20-min acquisition 90 min after injection. The Siemens Ordered Subset Expectation Maximization algorithm with Time-of-Flight (12 iterations, 21 subsets, no smoothing, resolution of full width at half maximum = 4.3 mm) was used to reconstruct all PET scans in high-resolution, as this reconstruction method maintains the natural variability in the data without smoothing^44^. Aβ PET images were spatially normalized using the CapAIBL software^45^. The standardized uptake value ratio (SUVR) was computed using the ratio of PET retention computed inside the neocortical Aβ CL mask and the whole cerebellum. The SUVR was then transformed into Aβ CL value using the published transform for [^18^F]NAV4694^14^.

The skull was stripped from tau images to remove off-target binding in non-brain regions. CT scans, acquired for attenuation correction, were used to generate a skull stripping mask using the FSL Brain Extraction Tool, that was subsequently transformed to the PET domain to extract the PET image brain^46^. Skull stripped images were manually checked for both registration and stripping faults. To evaluate the topography of [${}^{18}\mathrm{F}]$MK6240 bindings, tau-PET images were subject-wise, non-linearly normalized to the FSL MNI152 1 mm template using Advanced Normalization Tools after using the CT image as an intermediate step between the PET image and the template. The resultant tau PET images were each 158 × 198 × 158 voxels with 1 mm voxel size in all dimensions. Prior to input into the CNN, each tau-PET image was scaled to the range [0, 1], to examine the relative change of tau topography with Aβ burden and stabilize the training without exploding or vanishing gradients^47^. The [^18^F]-MK6240 scans were normalised using the cerebellar cortex, identified from the Automated Anatomical Labeling Atlas 3 (AAL3)^48^, to generate SUVR images analysed using voxel-wise general linear models for comparison with the CNN outcome.

Since tau is known to accumulate in medial and lateral temporal structures^6^, all brain slices are displayed as oblique axial slices throughout the results.

### Deep learning framework

The CNN accepted three-dimensional, skull stripped, normalized and scaled standardized uptake value (SUV) tau image data for each participant as input and, using the RMSprop optimizer, adjusted weights within the network to find the optimal mapping of the tau input to the Aβ CL. The CNN network structure of this study was influenced by the U-net architecture, which is widely used in image segmentation^16^. Since CNN model performance is sensitive to network structure and the associated parameters, several different CNN models were trained with 10 separate training/validation partitions (109 training sets, 12 validation sets; 10-fold cross-validation), selecting the model with the lowest root mean squared error (RMSE) across the partitions. More details of the selected CNN network structure and the training parameters are provided in Supplementary Sect. S.1.

All 10-fold cross-validation instances of the selected CNN model were evaluated on 13 separate, hold-out test sets. Out of the 10 instances of the selected model, the instance with the best performance, determined by the RMSE value evaluated using both validation and testing sets, was used for further analysis.

### Interpretation of CNN via saliency maps

After CNN training, the learned model requires interpretation to garner insight into how it maps the tau-PET topography to the associated Aβ burden. Saliency maps of equal dimension to the MNI normalized input tau images were generated for each subject^27^, using training, validation and testing datasets and across all ten cross-validation model instances. The saliency maps provide a measure of the importance of the voxels in the prediction of the output CL Aβ value. As saliency maps, being a voxel-wise measure, are visually noisy^26^, they were smoothed using a 2 mm FWHM Gaussian kernel before the analysis. Further details about saliency map computation are provided in Supplementary Sect. S.2.

### Comparison of CNN-based mapping with SUVR analyses

To evaluate the voxel-wise relationship between CNN-generated saliency values and Aβ CL values, GLM analyses were carried out using statistical parametric mapping (SPM) version 8. Saliency value was used as the dependent variable in GLM. Age and sex were identified as confounding variables and controlled. The following models were tested:

1. Tau-SUVR values are estimated by CL Aβ values, controlling for age and sex.
2. Saliency values are estimated by CL Aβ values, controlling for age and sex. The nonlinearity of the CNN method is encapsulated in the saliency maps.

SPM analysis (t-contrast) was carried out on both linear models to identify significant clusters with *p* < 0.05, controlling for multiple comparisons using the family-wise error (FWE) rate, with a minimum cluster extent of 200 voxels imposed.

### Interpretation of the identified saliency map clusters

The GLM analysis of the saliency maps identified statistically significant clusters. To determine the importance of best cross-validation instance clusters in the mapping between tau and Aβ CL value, each cluster was removed (‘occluded’) from the input images in turn, and new CNN outputs were predicted for all subjects. The importance of a cluster was assessed by quantifying the change in the coefficient of determination (*R*^2^) after its removal from the input data space. Out-of-sample *R*^2^ values were calculated on validation and testing datasets^49^. Since the CNN approach is a non-linear regression method, *R*^2^ has been used as a proxy value to evaluate this change of CNN outputs.

To evaluate the change of the importance of each cluster across the Aβ CL continuum, a ‘strength of association’ metric was introduced, quantifying the importance of each cluster and sub-cluster in mapping the corresponding Aβ CL level. Absolute errors were calculated between CNN outputs corresponding to full tau input images and occluded input tau images and these errors were smoothed with local weighted regression to generate the strength of association. To reduce the bias of the best cross-validation instance on the results, the mean strengths of association were calculated for the best cross-validation instance clusters using all the cross-validation instances. The identified salient clusters were divided into sub-clusters and the strengths of association of those sub-clusters were also analysed.

### Ethics approval

Ethics approval and consent to participate in this study were approved by the Austin Health Human Research Ethics Committee (HREC/18/Austin/201).

### Informed consent

This study was approved by the Austin Health Human Research ethics Committee (HREC/18/Austin/201) and given the retrospective nature of the study and the use of anonymized consented patient data under Austin HREC, requirements for informed consent were waived.

## Conclusion

A data-driven deep learning approach, unconstrained by standard definitions of pathological regions or reference regions, driven by relative spatial positioning rather than uptake levels, has revealed new relationships between tau topography and Aβ burden. This relationship does not start late in the natural history of AD, rather it occurs at minimal or low levels of Aβ burden. The differential importance of tau accumulation regions with the Aβ load may be considered as a proxy for longitudinal change and may provide insight into Alzheimer's disease evolution.

## Supplementary Information

Supplementary Information.

## Data Availability

The data that support the findings of this study are available from AIBL but restrictions apply to the availability of these data, which were used under license for the current study, and so are not publicly available. Data are however available from the authors upon reasonable request and with permission of AIBL (https://aibl.csiro.au/adni/index.html).
