# Supplementary material for: Mapping the association between tau-PET and Aβ-amyloid-PET using deep learning
Source: Sci Rep. 2022 Aug 30;12:14797. doi: 10.1038/s41598-022-18963-6 (PMC9427855; doi:10.1038/s41598-022-18963-6)
Supplement: Supplementary file 1 — Supplementary Information. [file 41598_2022_18963_MOESM1_ESM.docx]

**Mapping the association between tau-PET and Aβ-amyloid-PET using deep learning**

Gihan P. Ruwanpathirana^1,2^, Robert C. Williams^2^, Colin L. Masters^4,5^, Christopher C. Rowe^3,4,5^, Leigh A. Johnston^1,2^, Catherine E. Davey^1,2,*^

^1^ Department of Biomedical Engineering, The University of Melbourne, Melbourne, VIC, Australia.

^2^ Melbourne Brain Centre Imaging Unit, The University of Melbourne, Melbourne, VIC, Australia

^3^ Department of Molecular Imaging & Therapy, Melbourne, Austin Health, VIC, Australia

^4^ Florey Institute of Neuroscience and Mental Health, Melbourne, VIC, Australia

^5^ Florey Department of Neuroscience and Mental Health, The University of Melbourne, Melbourne, VIC, Australia

**SUPPLEMENTARY MATERIALS**

**S.1 Convolutional neural network (CNN) model**

An overview of the best performing CNN structure used in this study is given in Fig. S1, with five repeat convolution blocks, each containing two sequential convolutional layers to allow for different spatial filtering extents (3 x 3 x 3 kernel size, stride 1) with a leaky rectified linear (ReLU) activation function (slope of 0.01 at negative values), followed by a max-pooling layer (2 x 2 x 2 kernel size, stride 2) that identifies dominant features in the local feature map. In the first block, there are sixteen simple features across two convolutional layers within the block. The next convolutional block identifies combinations of the 16 simple features to make more complex features, allowing for 32 complex feature maps and continues to double after each block up to a maximum of 256 features. The final features are max-pooled and flattened before feeding to a fully connected layer of 1024 neurons, with a leaky ReLU activation, to estimate the Aβ CL output as a weighted sum of complex features.


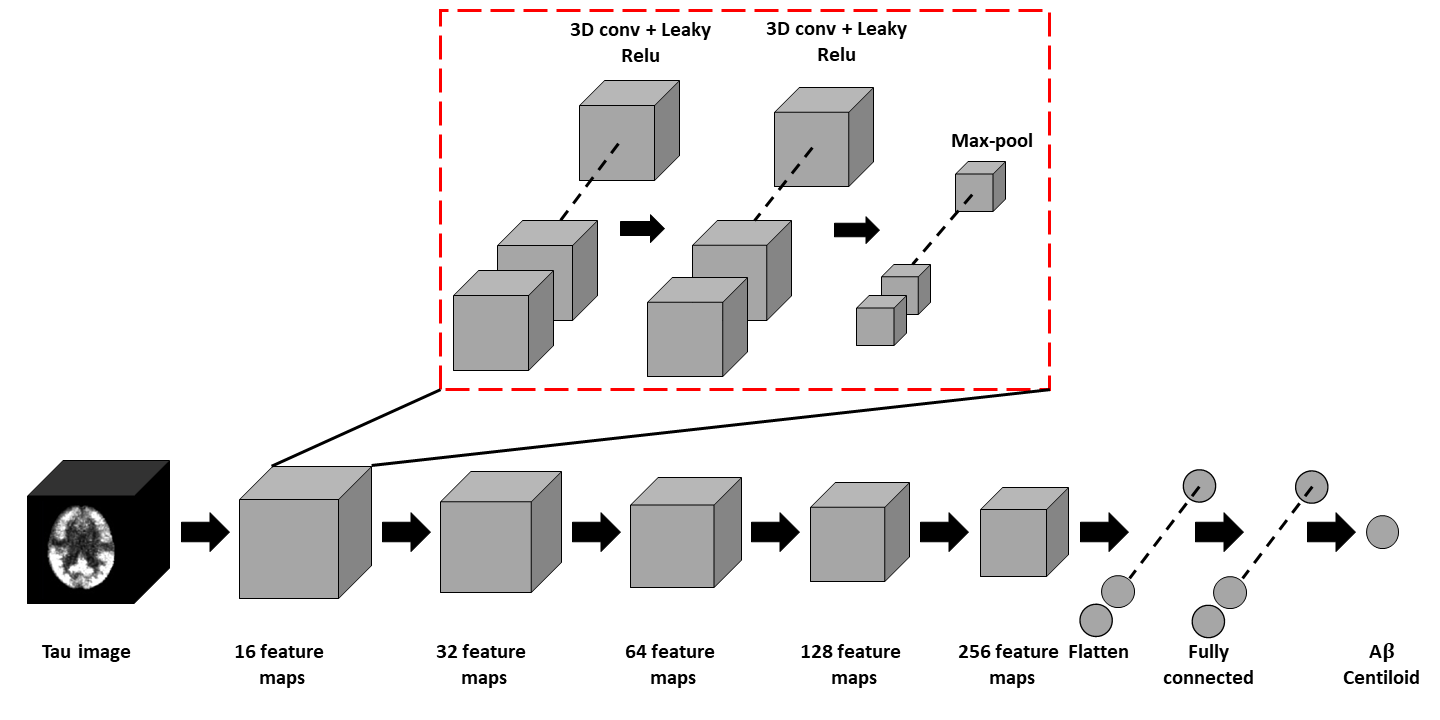


**Figure S1:** Overview of the CNN. There are five convolution blocks with the same configuration with two convolutional layers and one max-pooling layer. The red box shows an example of a convolution block. Each cube models a distinct feature map within the layer. Both convolutional layers in the block have the same number of feature maps, which are given below the corresponding convolutional block. Arrows show the propagation of information through the network layers. Feature maps at the end of the last convolution block are flattened and connected to a fully connected layer with 1028 neurons to output, the Aβ CL number.

Weights of the network were trained using backpropagation with a cost function defined as the root mean squared error (RMSE) between the measured Aβ CL and the CNN estimated CL. RMSprop optimiser with a decay of ${50}^{-6}$ was used to update the network weights while reducing the RMSE loss. The training was completed using 100 epochs with a constant learning rate of ${100}^{-6}$ and a mini-batch size of 16. The training epoch with the minimum RMSE value for the validation dataset was saved as an optimal point of training and used to carry out further analyses.

**S.2 Saliency maps**

To interpret how the optimal CNN maps an input tau image to an output Aβ CL, we generated a saliency map for each participant, assigning each voxel from the tau image a saliency score according to its contribution to the estimated Aβ CL^27^. Let’s consider given tau input image *I_0_* with CNN estimated Aβ CL, *S(I_0_)*. Saliency maps are supposed to give an importance value based on their impact on the *S(I_0_)*. Let’s consider a linear model for predicting the Aβ CL:

|  | $\text{S}\text{(I)}\text{ }\text{=}\text{ }\text{w}_{\text{ }}^{\text{T}}\text{I}_{\text{ }}\text{ }\text{+}\text{ }\text{b},$ | (1) |
| --- | --- | --- |

where the input tau image *I* is vectorised and *w* and *b* are the weight vector and the bias of the model, respectively. Therefore, we can see that elements of the *w* give the importance of corresponding voxel of input image *I* in estimating the Aβ CL, *S(I)*. The relationship between *I* and *S*(*I*) is typically nonlinear in a CNN, but can be considered approximately linear in the neighborhood of a given input image, *I_0_*, such that

|  | $\text{S}\left( \text{I}_{\text{0}} \right)\text{ }\text{≈}\text{ }\text{w}_{\text{ }}^{\text{T}}\text{I}_{\text{0}}\text{ }\text{+}\text{ }\text{b}_{\text{ }},$ | (2) |
| --- | --- | --- |

where *w* and *b* are the weight vector and bias value of the proposed model, respectively. The weight vector *w* for a given tau input image *I_0_* is calculated as:

|  | $w=\frac{\delta S\left( I_{0} \right)}{\delta I_{0}}$ | (3) |
| --- | --- | --- |

Therefore, the partial derivative of the CNN output with respect to the input tau image gives the weight vector, which can be transformed to the saliency map. That is, the saliency of each voxel’s tau value quantifies its importance in the tau-to-A$\beta$CL mapping.


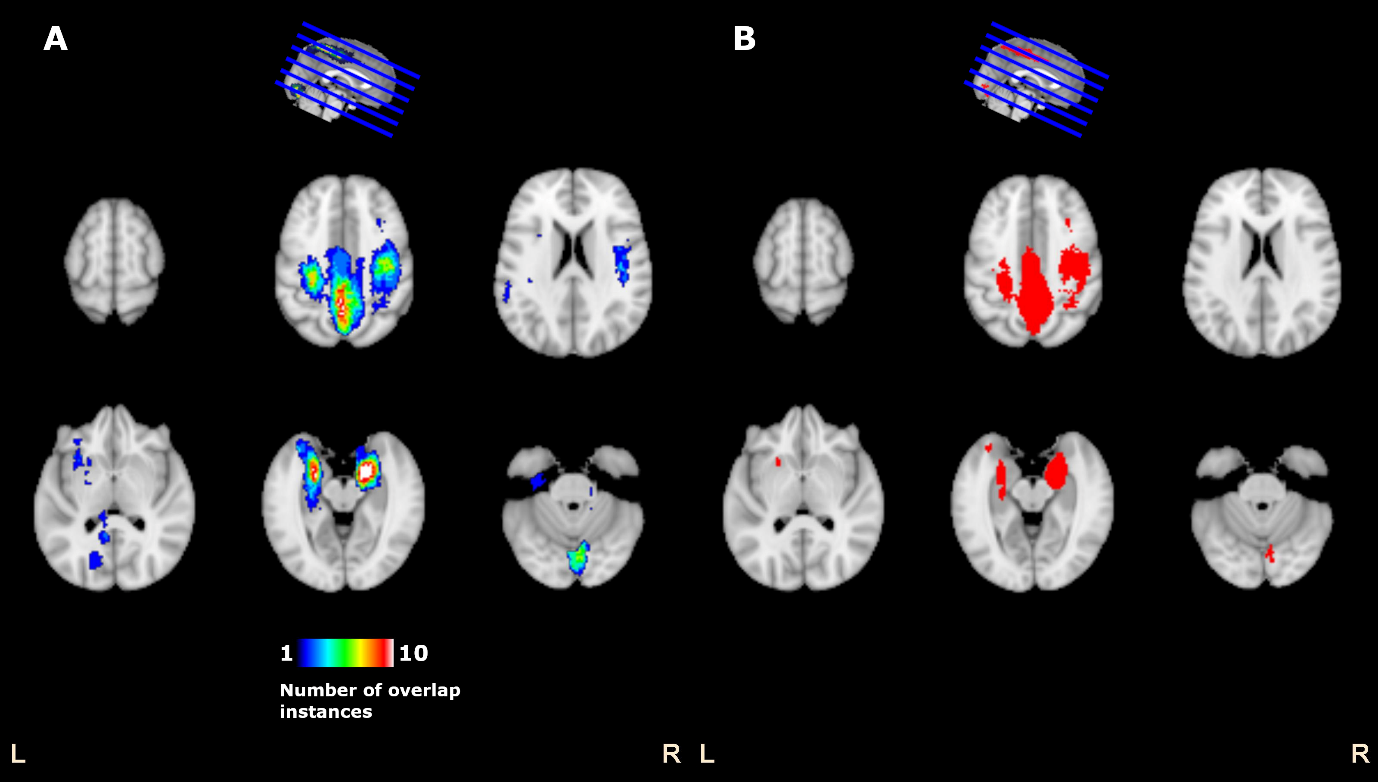


**Figure S2:** Comparison between CNN-based salient clusters which are associated with Aβ CL, as identified by the GLM saliency analysis of **(A)** all 10-cross validation instances and **(B)** the selected cross-validation instance of the best performing CNN model, picked after comparing several different CNN models. Voxel values in panel **A** denote the number of cross-validation instances that identified those voxels as significant.

**Table S1:** Performance measures of the CNN model with the best average root mean square error (RMSE) across the validation datasets in each of the cross-validation 10-folds. Performance measures, RMSE and *R*^2^, are shown for training, validation and test datasets, across all 10-folds, with format mean (SD). *R^2^* is a measure of goodness-of-fit which represents the proportion of the variance of Aβ CL explained by the CNN model.

| **Performance measure** | **Training**  **Mean (SD)** | **Validation**  **Mean (SD)** | **Testing**  **Mean (SD)** |
| --- | --- | --- | --- |
| RMSE | 23.21 (8.07) | 30.10 (10.23) | 34.09 (4.95) |
| *R*^2^ | 0.86 (0.10) | 0.75 (0.18) | 0.72 (0.08) |

**Table S2:** Performance of the best CNN model on each of 10-folds of the cross-validation. The highlighted instance was considered optimal based on having the best performing and consistent RMSE across training, validation and test phases and used for further analysis.

| **Cross-validation model number** | **Training RMSE** | **Validation RMSE** | **Testing RMSE** | **Training R^2^** | **Validation R^2^** | **Testing R^2^** |
| --- | --- | --- | --- | --- | --- | --- |
| 1 | 20.75 | 44.29 | 36.91 | 0.90 | 0.44 | 0.68 |
| 2 | 32.58 | 21.29 | 43.78 | 0.76 | 0.86 | 0.54 |
| 3 | 20.38 | 33.42 | 28.22 | 0.90 | 0.82 | 0.82 |
| 4 | 21.81 | 34.19 | 32.8 | 0.89 | 0.71 | 0.75 |
| 5 | 28.49 | 18.60 | 34.59 | 0.82 | 0.89 | 0.72 |
| 6 | 11.72 | 15.08 | 29.93 | 0.97 | 0.96 | 0.79 |
| 7 | 39.05 | 22.56 | 39.15 | 0.64 | 0.89 | 0.64 |
| 8 | 17.09 | 38.6 | 28.76 | 0.94 | 0.48 | 0.81 |
| 9 | 22.84 | 42.32 | 35.68 | 0.88 | 0.63 | 0.70 |
| 10 | 17.41 | 30.62 | 31.12 | 0.93 | 0.81 | 0.77 |
| Mean (SD) | 23.21 (8.07) | 30.10 (10.23) | 34.09 (4.95) | 0.86 (0.10) | 0.75 (0.18) | 0.72 (0.08) |
